# Ditopic Aza-Scorpiand Ligands Interact Selectively with ds-RNA and Modulate the Interaction upon Formation of Zn^2+^ Complexes

**DOI:** 10.3390/molecules26133957

**Published:** 2021-06-28

**Authors:** Lluís Guijarro, Álvaro Martínez-Camarena, Javier U. Chicote, Antonio García-España, Enrique García-España, Mario Inclán, Begoña Verdejo, Jorge González-García

**Affiliations:** 1 Instituto de Ciencia Molecular (ICMol), Departamento de Química Inorgánica, Universidad de Valencia, C/Catedrático José Beltrán 2, 46980 Paterna, Spain; lluis.guijarro@uv.es (L.G.); alvaro.martinez@uv.es (Á.M.-C.); mario.inclan@uv.es (M.I.); 2 Institut d’Investigació Sanitària Pere Virgili (IISPV), Centro de I+D+I en Nutrición y Salud Avda. de la Universitat 1, 43204 Reus, Spain; jugarte@piushospital.cat (J.U.C.); antoniogem85@gmail.com (A.G.-E.); 3 Pathology Unit, Joan XXIII University Hospìtal, C/Dr. Mallafrè Guasch 4, 43005 Tarragona, Spain

**Keywords:** aza-macrocycle, DNA and RNA duplexes, zinc complex

## Abstract

Nucleic acids are essential biomolecules in living systems and represent one of the main targets of chemists, biophysics, biologists, and nanotechnologists. New small molecules are continuously developed to target the duplex (ds) structure of DNA and, most recently, RNA to be used as therapeutics and/or biological tools. Stimuli-triggered systems can promote and hamper the interaction to biomolecules through external stimuli such as light and metal coordination. In this work, we report on the interaction with ds-DNA and ds-RNA of two aza-macrocycles able to coordinate Zn^2+^ metal ions and form binuclear complexes. The interaction of the aza-macrocycles and the Zn^2+^ metal complexes with duplex DNA and RNA was studied using UV thermal and fluorescence indicator displacement assays in combination with theoretical studies. Both ligands show a high affinity for ds-DNA/RNA and selectivity for ds-RNA. The ability to interact with these duplexes is blocked upon Zn^2+^ coordination, which was confirmed by the low variation in the melting temperature and poor displacement of the fluorescent dye from the ds-DNA/RNA. Cell viability assays show a decrease in the cytotoxicity of the metal complexes in comparison with the free ligands, which can be associated with the observed binding to the nucleic acids.

## 1. Introduction

Nucleic acids (DNA and RNA) play essential roles in storing genetic information and templating the formation of proteins in living systems. Modulation of the nucleic acid metabolism, including DNA replication, translation, and transcription, by synthetic or natural molecules has been the basis of current clinical chemotherapy. Initially, DNA became the most interesting biomolecule to target, being *cis*-diamminedichloridoplatinum(II) (cisplatin), the first anticancer molecule used in clinics. The action mechanism of cisplatin involves DNA binding, halting of replication, and induction of apoptosis [1]. In order to treat human diseases such as microorganism infections and genetic disorders, which rank among the most difficult diseases to tackle, the paradigm has recently shifted to RNA targeting with small molecules [2,3,4,5].

To overcome the limitations of nucleic acid agents such as poor solubility, side effects, dose-limiting toxicity, distribution in tissues, or acquired resistance, several approaches have emerged combining chemo- and radiotherapies with more effective drug delivery systems [6,7]. A promising strategy is the development of new stimuli-responsive nucleic acid binders in which a given stimulus promotes or hinders the nucleic acid binding to the molecule and, therefore, modulates the biological activity associated with the nucleic acid metabolism. Mascareñas, Vazquez, and co-workers have finely designed several stimuli-responsive systems based mainly on synthetic polypeptide helices that bind to DNA upon light irradiation and metal coordination and are compiled in a recent review [8]. Among the triggering stimuli, metal coordination is one of the most challenging strategies due to the abundance of metal ions in cells that can occupy the coordination sites of the synthetic systems and hamper the envisaged DNA interaction process. In this regard, Peacok’s team reported two short peptides connected by a bipyridine or a terpyridine unit that undergo conformational changes upon Zn^2+^ or Cu^2+^ coordination, prompting their interaction with double-stranded DNA (ds-DNA) [9]. An interesting system described by Learte-Aymami et al. is composed of a peptide moiety containing histidine units, which is unable to bind DNA because of the unstructured nature in solution, but Pd^2+^ coordination to the nitrogen atoms of the histidines promotes its folding, adopting a helical conformation with a concomitant high ds-DNA binding [10]. In contrast, metal coordination can also inhibit the binding to nucleic acids. For instance, Futaki and co-workers described as Co^2+^ coordination to some peptide dimeric systems containing iminodiacetic acid groups decreases their structural helical arrangement reducing thereby, their interaction with DNA [11]. In this line, our group is devoted to the development of polyamine-based systems because they present interesting structural features for modulating the binding of anionic species, in particular, (*i*) the significant changes in net charge within short pH ranges [12] and (*ii*) the ability to strongly coordinate metal ions [13,14].

Regarding nucleic acids, we have developed a series of scorpiand-like ligands containing a macrocyclic core and a pendant arm with an aromatic moiety, which display an open extended or a closed conformation depending on the pH and metal coordination [15]. While the open extended conformation of the scorpiand favors an intercalative binding to DNA of condensed aromatic groups such as anthracene or pyrene introduced in the pending arm, the closed conformation of the ligand achieved by metal binding or by an increase in pH blocks the intercalation [16,17]. In addition to metal coordination, we have explored the ability of polyprotonable ligands to modulate the binding to either DNA or RNA by changing the pH. A change in the pH from 7.4 to 5.0 increases the net charge of the ligand favoring the interaction with RNA over DNA by means of electrostatic interactions [18,19].

**Chart 1:**
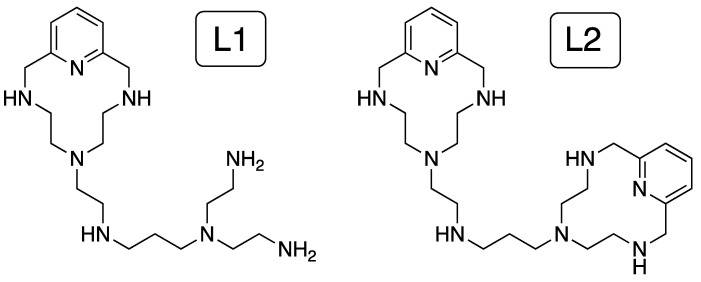
Ligands (**L1** and **L2**) studied in this work.

Herein we explored the ability of two ligands containing two different coordinating sites to bind double-stranded DNA and RNA (ds-DNA/RNA) differing in the conformation, B- and A-type, respectively, either as free ligands or coordinated to Zn^2+^ ions (see Chart 1). We studied the acid-base behavior and metal coordination by potentiometry to determine the net charge and characterize the Zn^2+^ complexes formed in solution. Then, we combined thermal stabilization experiments, fluorescent indicator displacement assays, and theoretical multiscale studies to investigate the binding of the ligands and their corresponding Zn^2+^ complexes to ds-DNA/RNA. Finally, the cell viability was assessed for the ligand and their corresponding metal complexes to determine the biological activity.

## 2. Results

### 2.1. Characterization of **L1**–**L2** and Their Zn^2+^ Complexes

#### 2.1.1. Speciation in Solution of **L1**–**L2**

**Figure 1:**
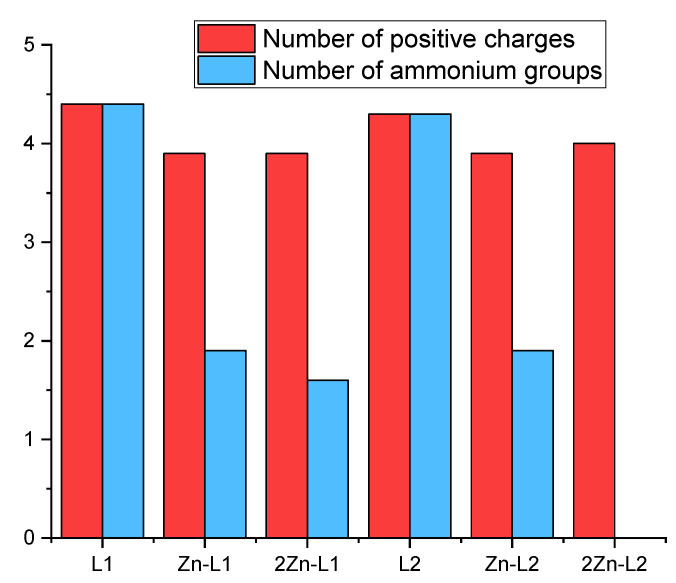
Average number of positive charges and number of ammonium groups at pH 7.4 (L = 1 × 10^−5^ M, Zn^2+^ = 1 × 10^−5^ M and 2 × 10^−5^ M for Zn-L and 2Zn-L complexes, respectively) derived from the values of stability and protonation constants previously obtained by potentiometric titrations in aqueous solution at 298.1 K using NaCl 0.15 M as supporting electrolyte.

The knowledge of the species prevailing in aqueous solution and their protonation state is key to understand the effect of electrostatic forces and hydrogen bonding in host-guest complexation events. However, these parameters are often difficult to determine in water due to solubility issues. In our study, the ligands **L1** and **L2** (described and characterized previously in reference [20]) are totally water soluble within the pH range, and thus, we studied the acid-base and metal coordination behavior of both **L1** and **L2** by pH-metric titrations. Stepwise protonation constants of the ligands determined in 0.15 M NaCl at 298.1 K, as well as their distribution diagrams, are collected in Supplementary Materials (Table S1 and Figure S1) in ESI. As discussed in reference [20], **L1** and **L2** present five protonation steps in the pH range of study, being their net charge at physiological pH 7.4, calculated from the distribution diagrams, +4.4 and +4.3, respectively (Figure 1).

Then, the formation of Zn^2+^ complexes with **L1** and **L2** was studied in solution by potentiometric measurements [20]. The stability constants provided by the pH-metric titrations (Table S2) allowed us to establish the distribution diagrams gathered in Figures S2 and S3. Such representations show the formation of the binuclear species Zn_2_L^+^, Zn_2_L(OH)^3+^ and Zn_2_L(OH)_2_^2+^ for both ligands in a 2:1 Zn^2+^:L molar ratio (Figure S3). The net charges of the complexes at pH = 7.4, calculated for 1:1 and 2:1 Zn^2+^:L mole ratios from the distribution diagrams, are just a little bit lower than those of the free ligands. (see Figure 1). However, the coordination of the Zn^2+^ ions, particularly in the case of the binuclear complexes, drastically reduces the number of ammonium groups present at pH 7.4, altering, therefore, the possible pattern of hydrogen bonds of the ligand with nucleic acids (Figure 1). The stability constant values for the formation of the mononuclear species of both ligands are within the same range, suggesting that the entry of the metal is occurring in the common structural core, which is the macrocycle. The lower value of the stability constant obtained for the entry of the second metal for **L1** (7.89) in comparison with **L2** (11.70) suggests a stronger interaction of the metal ion with the nitrogen atoms of the macrocycle than the nitrogen atoms of the acyclic part in **L1**.

#### 2.1.2. QM Modeling of the Zn^2+^ Complexes

**Figure 2:**
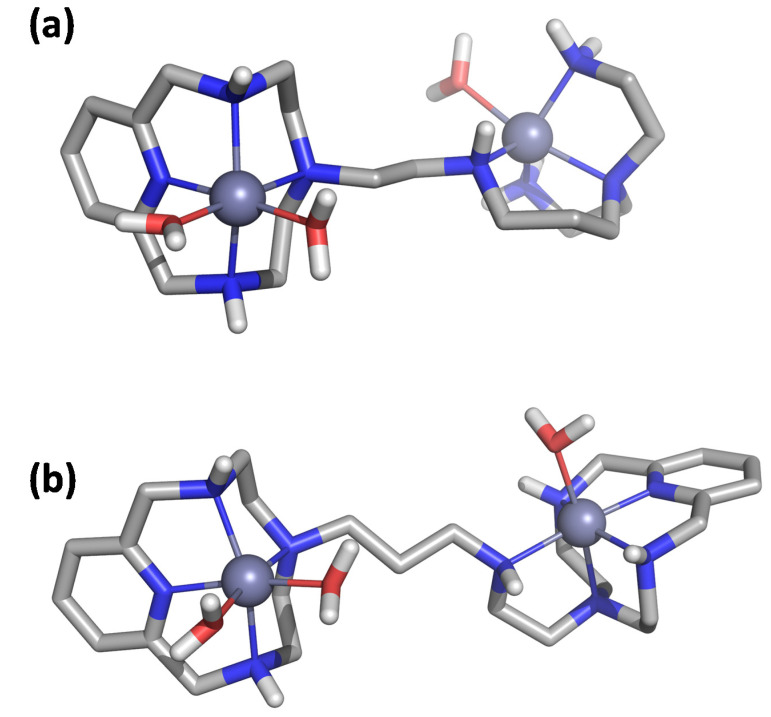
Visualization of the minimum energy structure optimized by DFT for the complex Zn_2_**L1**^4+^ (**a**) and Zn_2_**L2**^4+^ (**b**).

We initially optimized the geometry of the Zn^2+^ binuclear complexes of both ligands using DFT to afford the minimum energy conformers shown in Figure 2 (structural coordinates are collected in Tables S3 and S4). The metal ions in Zn_2_**L1**^4+^ show different coordination modes depending on the coordination site. The Zn^2+^ ion at the macrocyclic site is coordinated by the four nitrogen atoms of the macrocyclic moiety and by two water molecules in a distorted octahedral geometry, while the second metal ion is coordinated by the four nitrogen atoms of the acyclic side chain and a water molecule with a trigonal bipyramid geometry (Figure 2A). In contrast, in the binuclear Zn_2_**L2**^4+^ complex, both metal centers present an octahedral coordination geometry in which each metal ion is coordinated by the four nitrogen atoms of each macrocyclic moiety. However, the coordination sphere of each Zn^2+^ is completed differently. In the metallic center separated from the amine group of the linker by an ethylenic chain, besides the nitrogens of the macrocycle, the metal ion is coordinated by a water molecule and the amino group of the linker. On the other hand, the second metal ion completes its coordination with just one water molecule (Figure 2B).

### 2.2. Interaction Studies of Ligands to Double-Stranded Nucleic Acids

#### 2.2.1. Thermal Denaturation Experiments

We investigated the interaction of the ligand with ds-DNA/RNA by means of thermal stabilization assays. Double-stranded oligomers are held together by hydrogen bonds, which can be dissociated upon heating to provide single-stranded oligomers. During the thermal denaturation process, a melting temperature or transition temperature (*T*_m_) can be determined as the temperature in which half of the DNA strands are in the random coil or single-stranded state. High CG contents or non-covalent interactions with small molecules usually stabilize the double-stranded helical structure. The variation between the melting temperature of the free ds-DNA/RNA and of the adducts formed with small molecules (Δ*T*_m_) is a qualitative parameter for the interaction. The Δ*T*_m_ values for the interaction of **L1** and **L2** with a ds-DNA model of B-type conformation (calf thymus DNA/ctDNA) and a ds-RNA model (poly A-poly U) with an A-form duplex are gathered in Table S5 for different molar ratios (r = [ligand]/[polynucleotide]).

**Figure 3:**
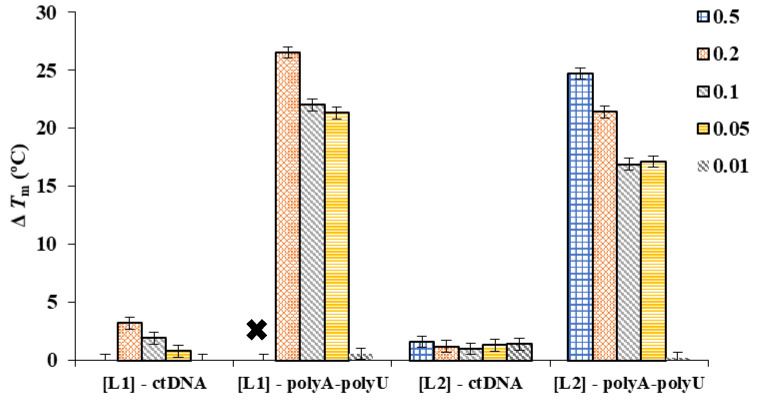
Δ*T*_m_ values determined for the interaction of **L1** and **L2** toward ctDNA and poly A-poly U at different r = [ligand]/[polynucleotide]. Each bar stands for an r value, as is described in the legend. The “**×**” indicates a Δ*T*_m_ value too high to be determined.

Interaction of **L1**/**L2** with ds-DNA and ds-RNA leads to thermal stabilization of both double helixes (see Figure 3). The Δ*T*_m_ values obtained for ctDNA ranged between 0.1 and 3.2 °C, while for poly A-poly U were higher than 15 °C, indicating a stronger and selective interaction with the RNA duplex model. The strong interaction of these polyamine-based ligands with poly A-poly U is probably due to the A-type conformation of this ds-RNA, which exposes the negative charges of the nucleic acid outwards the helix and allows a more efficient interaction with the positively charged ligands than in the case of the B-type helix adopted by ds-DNA. The higher Δ*T*_m_ values achieved for **L1** and **L2** at high r = [ligand]/[polynucleotide] ratios (r = 0.2 and 0.5) are among the largest ones observed in the literature for these duplex models [21].

#### 2.2.2. Fluorescent Indicator Displacement Assays

**Chart 2:**
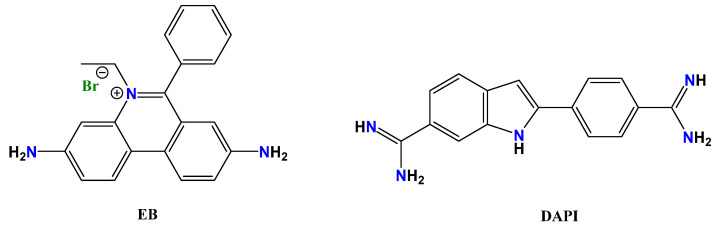
Fluorescent dyes used for displacement assays, ethidium bromide (EB) and DAPI.

Once established the strong interaction of **L1** and **L2** with duplexes and, in particular, with ds-RNA, we used fluorescence indicator displacement assays to estimate the binding affinity. Indirect measurements were employed due to the non-emissive character of both ligands, as well as to the fact that their absorption bands overlap with those of the nucleic acids, which hampers to perform direct fluorimetric and UV-Vis titrations. In addition, we used a well-established intercalative dye (ethidium bromide, EB, Chart 2) and a minor groove binder (DAPI) to study the binding mode. Both dyes are poorly emissive in solution but become highly emissive when bound to duplex DNA/RNA.

##### EB Displacement Assays

**Figure 4:**
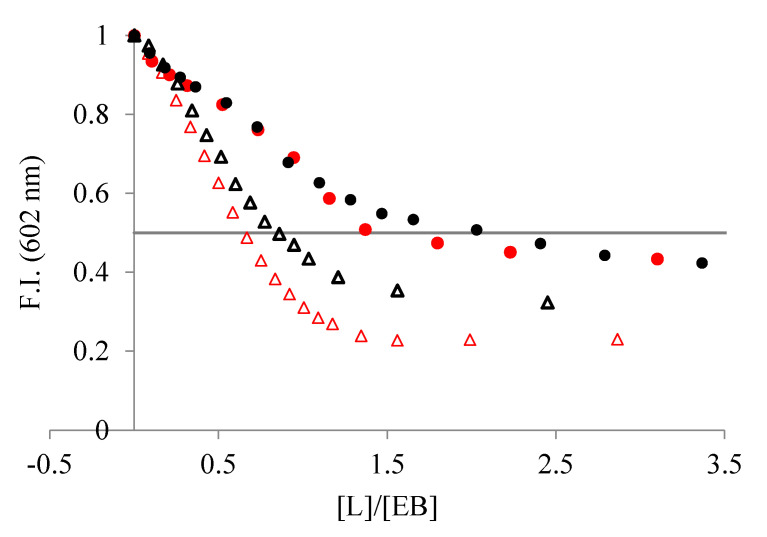
Changes in fluorescent emission obtained from EB displacement assays of (●) ctDNA and (Δ) poly A-poly U, 3–4 × 10^−5^ M, when titrated with (red) **L1** and (black) **L2** (NaCac 50 mM, pH 7.4). The line represents the emission decrease by 50% with respect to the initial value.

The addition of the ligands to a solution of duplex DNA/RNA slightly saturated with the intercalator EB induced a decrease in the fluorescence emission of the EB dye (Figure 4) because EB is displaced from the hydrophobic nucleic acid matrix to the solution. As shown in Figure 4, the addition of the ligands yields a larger decrease in emission intensity when interacting with poly A-poly U than with ctDNA. Since a decrease in emission is directly associated with the extent of binding, we can conclude that **L1** and **L2** interact stronger with ds-RNA than with ds-DNA.

From the displacement assay isotherms, we calculated the IC_50_ values to estimate the binding affinities, in which the IC_50_ is the [ligand]/[DNA] ratio corresponding to the emission decrease by 50% with respect to the initial value (Figure 4). Both ligands show lower IC_50_ values for poly A-poly U than for ctDNA, indicating a higher affinity for the ds-RNA (see Table 1). Moreover, **L1** binds to both duplexes stronger than **L2,** suggesting that it has a structure better suited to interact with the double-stranded nucleic acid structure.

The values of the affinity constants (K_app_) can be calculated by taking into account such IC_50_ values and the affinity constants for the interaction of EB with ctDNA and poly A-poly U under the same experimental conditions (pH 7.0, log K_app_ = 10^6^) [22]. The affinity constants range between K_app_ ~ 10^5^–10^6^ M^−1^ (see Table 1).

##### DAPI Displacement Assays

**Figure 5:**
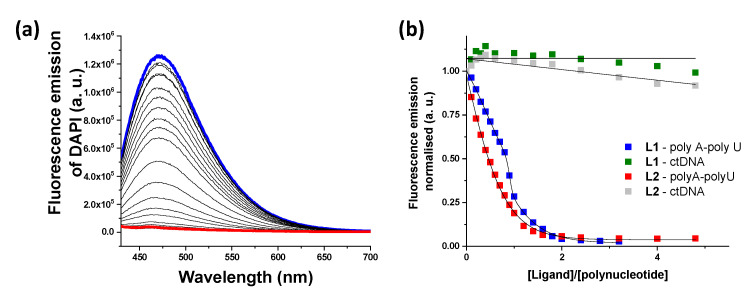
(**a**) Changes in fluorescent emission obtained from DAPI displacement assays with poly A-poly U and **L1** (blue line, initial spectrum and red line, final spectrum). (**b**) Plot of the fluorescence emission vs. ratio in the DAPI displacement assays.

In order to find out whether the ligands displayed an intercalative or a minor groove binding mode, displacement measurements were performed using DAPI as a fluorescent dye (Figure 5). A decrease in DAPI emission was only observed for ds-RNA, suggesting that both ligands bind to the minor groove of poly A-poly U and are not able to significantly interact with ds-DNA. The values of IC_50_ are collected in Table 1 together with the values obtained from the EB assay. The values obtained from the DAPI displacement assay for ds-RNA are within the same range as those obtained from the EB assay (see Table 1).

#### 2.2.3. Molecular Modeling

**Figure 6:**
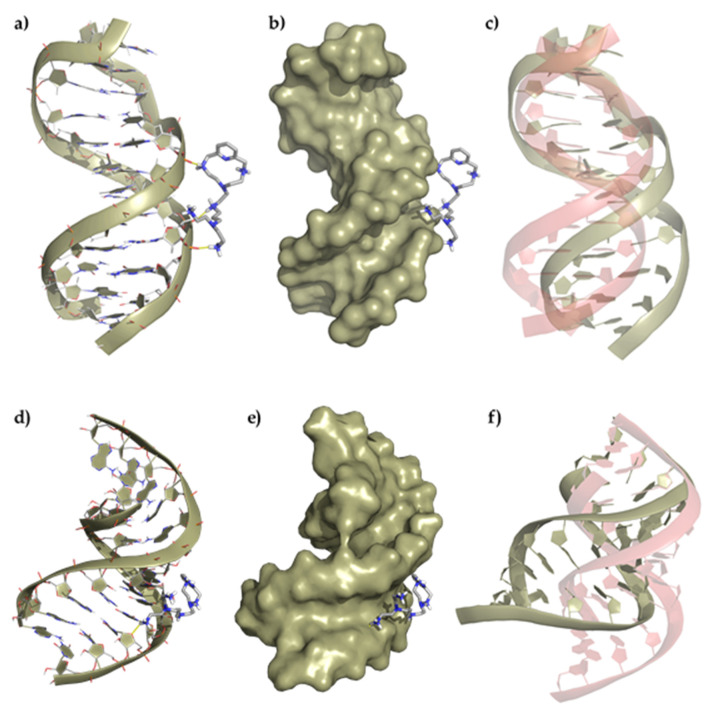
Top panel, representation of (**a**) the interaction of **L1** with the ds-DNA, (**b**) the polynucleotide surface, and (**c**) deviation of the duplex DNA structure after interaction regarding its original conformation (represented in red). Bottom panel, representation of (**d**) the interaction of **L1** with the duplex RNA, (**e**) the polynucleotide surface, and (**f**) deviation of the ds-RNA structure after interaction regarding its original conformation (represented in red).

Once we assessed the interaction of the ligands with duplexes by means of UV melting and fluorescent indicator displacement assays, multiscale QM/MM-MD studies were conducted to obtain insights into the binding mode. The structures shown in Figure 6 (Supplementary Materials Figures S4 and S5) show the minimum energy conformers determined for the interaction of the ligands with both the DNA and RNA duplexes through the minor groove. Strikingly, both ligands bind externally to the duplex DNA by means of electrostatic interactions with the phosphate backbone while they are inserted into the minor groove of RNA duplex model forming a larger number of electrostatic and hydrogen bond contacts. The ability to bind in the minor groove of RNA can be ascribed to the ligand shape that fits better in the shallow and wide minor groove of RNA ((3.70 Å deep and 10.36 Å wide) than in the narrower and deeper one of DNA (5.96 Å deep and 5.37 Å wide).

A detailed analysis of the minimum energy conformers of **L1** with ds-DNA shows the formation of three salt bridges between the oxygen atoms of the phosphate groups and some of the protonated amines of the ligand (d_O···H–N_ = 1.64–1.76 Å), which slightly distorts the helical structure. On the other hand, the adduct formed between **L1** and ds-RNA gives rise to minimum energy conformers stabilized by the formation of a larger number of salt bridges between the oxygens of the phosphate groups and some of the protonated amines of the ligand (d_O···H–N_ = 2.08 Å), which has a greater impact on the RNA structure. The insertion of **L1** into the minor groove duplex bends the structure, forming an even wider and deeper minor groove (amplitude widens from 10.36 to 17.61 Å, and the depth decreases from 3.70 to 2.73 Å). A similar interaction is observed for the ligand **L2** and the DNA/RNA duplexes (Figures S4 and S5).

### 2.3. Metal-Induced Modulation of RNA Binding

Once determined the strong interaction and high-affinity constants of **L1**–**L2** toward ds-DNA/RNA, we explored the binding of the metal complexes with ds-RNA. Both ligands form mono- and binuclear metal complexes with Zn^2+^ at pH 7.4 depending on the molar ratio M:L (see Figures S2 and S3). We envisaged that the metal complexes of the ligands would induce a structural arrangement and/or will disable the ammonium groups for electrostatic contacts with ds-RNA because of their participation in the metal coordination.

#### 2.3.1. Thermal Denaturation Experiments of Metal Complexes

We studied the interaction of the mono- and binuclear Zn^2+^ complexes of **L1**–**L2** toward poly A-poly U by UV thermal denaturation experiments, and the Δ*T*_m_ values are collected in Table 2. In a previous work [20], we showed the formation of the mononuclear species ZnH_2_L^4+^ at pH 7.4 for both ligands at 1:1 molar ratio (M:L) while at 2:1 molar ratios, **L2** forms the species Zn_2_L^4+^ and **L1** presents a mixture of species, being predominant the species Zn_2_L^4+^ (~ 80% Zn_2_L^4+^, 15% Zn_2_HL^5+^ and free Zn^2+^) [20]. Therefore, we assume that the species interacting with ds-RNA will be ZnH_2_L^4+^ and Zn_2_L^4+^ for a 1:1 and 2:1 molar ratio, respectively. Interestingly, the coordination of the first Zn^2+^ ion is accompanied by a large reduction in the Δ*T*_m_ values, while the coordination of the second Zn^2+^ ion either induces a small stabilization or even a slight destabilization.

#### 2.3.2. Fluorescence Displacement Studies of Metal Complexes

**Figure 7:**
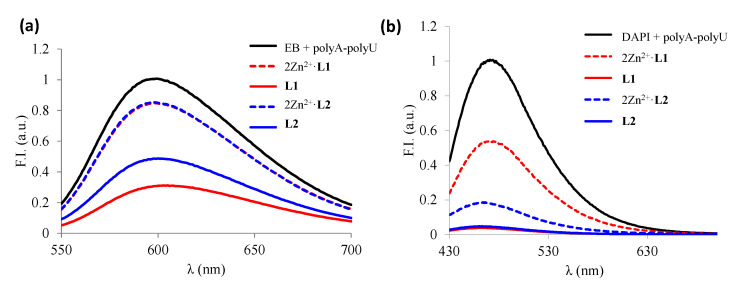
Changes in fluorescence spectra obtained from EB (**a**) and DAPI (**b**) displacement assays under the addition of free ligands and the corresponding 2:1 Zn^2+^:L complexes.

Once determined the ds-RNA stability with the **L1**–**L2** Zn^2+^ metal complexes, we evaluated the affinity of the binuclear species (Zn_2_L^4+^) toward ds-RNA by using the fluorescent indicator displacement assays with EB and DAPI. The displacement of the binuclear species depended on the fluorescence dye used (see Figure 7). The addition of the binuclear Zn^2+^ complexes released only 20% of the EB bound to poly A-poly U whereas they displaced 50% and 80% of the DAPI, respectively, indicating that the Zn_2_L^4+^ species interacts mostly through the minor groove of poly A-poly U. Nevertheless, both ligands displace completely both indicators from ds-RNA, indicating the larger affinity of the ligands in comparison with their metal complexes.

#### 2.3.3. Molecular Modeling

**Figure 8:**
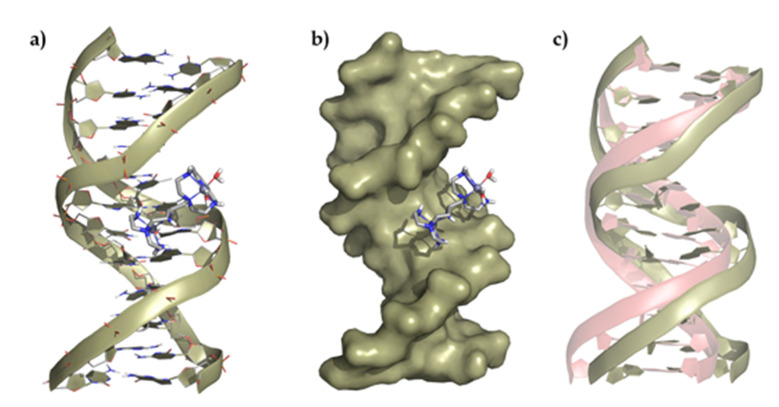
Representation of (**a**) the interaction of Zn_2_**L1**^4+^ with the ds-DNA model, (**b**) the polynucleotide surface, and (**c**) deviation of the ds-DNA structure after interaction regarding its original conformation (represented in red).

Multiscale QM/MM-MD methods were employed to generate a model for the interaction of the Zn^2+^ binuclear complexes with the DNA/RNA duplexes (Figure 8 and Supplementary Materials Figures S6–S8). The binuclear Zn_2_L^4+^ complexes of both ligands show a similar binding mode toward the ds-DNA and RNA models characterized by the interaction of the complexes through the major groove of the nucleic acid models. Interestingly, the low flexibility of the metal complexes, together with their limited capacity to form hydrogen bonds and to interact electrostatically, compared to the free ligands, leading to the formation of adducts with a smaller number of contacts and located more externally in the groove than the ligands alone. Therefore, the interaction of the Zn^2+^ complexes with the duplexes is weaker than that of the free ligands, as supported by the denaturalization and displacement studies.

### 2.4. Viability Assays of the Ligands and the Metal Complexes

**Figure 9:**
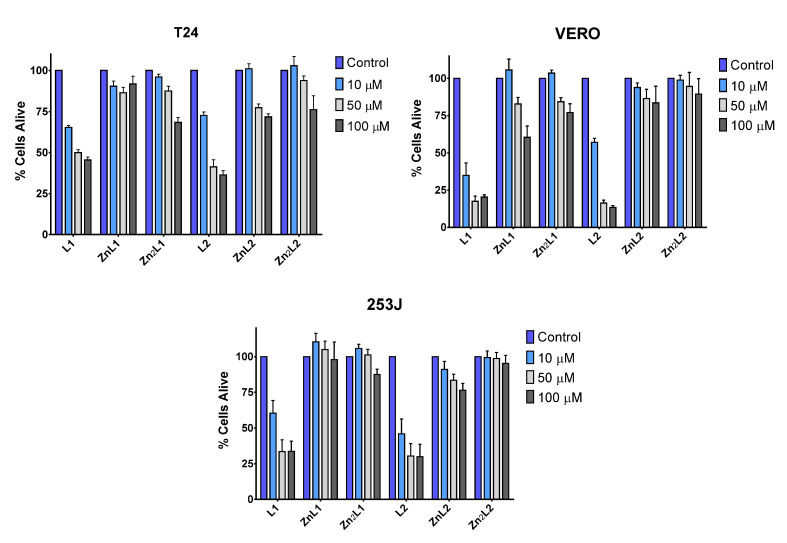
Low toxicity of **L1** and **L2** metal complexes. Toxicity of **L1**, **L2** and their metal complexes in mammalian cell lines. T24, VERO, and 253J cell lines were treated with 10, 50, and 100 μM of the compounds for 48 h to assess their toxicity based on the cell viability of the cell lines. The data are two independent experiments performed in triplicate.

Before determining the interaction of the ligands and their Zn^2+^ complexes with DNA and RNA, we investigated the effects of **L1**, **L2,** and their Zn complexes in cell viability. Two bladder cancer cell lines (T24 and 253J) with different mutations in the tumor suppressor genes p53 and RB1 and a human fibroblast cell line (VERO) were treated with different concentrations of ligands and complexes (Figure 9). While, in all cell lines, **L1** or **L2** produce a diminution of cell viability, which was maximal at higher doses, **L1** or **L2** metal complexes did not affect the cellular viability at 10 μM and always less than the uncoordinated ligands at higher doses (Figure 9). This result, in agreement with the biophysical studies, suggests that metal coordination strongly influences the cytotoxic activity of these ligands.

## 3. Discussion

The coordination with Zn^2+^ and protonation degree of two polyamine-based ligands were investigated by potentiometry. **L1** contains only one macrocyclic core with a pendant polyamine acyclic chain, while **L2** contains two macrocyclic cores connected by an ethylenaminopropyl linker. Both ligands form stable binuclear complexes with Zn^2+^ along all the pH range studied. Such Zn^2+^ binding leads to a reduction in the net charge of the systems due to the participation of the nitrogen atoms in the coordination of the Zn^2+^ ions. Theoretical studies afforded the minimum energy conformers of the metal complexes. **L2** presents octahedral geometry for both metal centers in the binuclear Zn^2+^ complex ([Zn_2_**L2**]^4+^ whereas **L1** shows one metal center with an octahedral geometry corresponding to the macrocyclic core and a trigonal bipyramid geometry for the acyclic core. Both ligands show solvent molecules completing the metal ion coordination sphere, which can potentially coordinate the bases and phosphate of DNA/RNA.

Then, the binding ability of both ligands was assessed by UV melting experiments and fluorescent displacement assays, which show a large stabilization effect and high affinity to RNA duplexes for both ligands. The higher Δ*T*_m_ values and lower IC_50_ values from the FID of **L1** can be associated with its higher flexibility in comparison with the more rigid structure of **L2** with two macrocyclic cores. The higher flexibility of **L1** facilitates adopting a conformation in a solution that maximizes the interaction contacts with the nucleic acids. Interestingly, FID assays using the minor groove binder DAPI show a displacement only in ds-RNA, suggesting that both ligands interact through this binding mode with RNA. These studies are supported by molecular modeling, which highlights the more external interaction of both ligands to the B-type conformation of ds-DNA in comparison with the minor groove binding to the A-type conformation of the ds-RNA model.

When forming the binuclear Zn^2+^ complex, the thermal denaturation and FID studies show a negligible stabilization and lower indicator displacement, indicating the modulation of the interaction to ds-RNA upon Zn^2+^ coordination. Moreover, theoretical calculations show a lower number of contacts with duplexes because the nitrogen atoms of the ligands are involved in the metal coordination.

To obtain some insights into the biological activity, the cell viability was assessed by means of MTT assay with three cell lines and show the same cytotoxic modulation as the biophysical studies. The free ligands show high cytotoxicity toward all the cell lines while the metal coordination increases the viability of cells. This effect can be attributed to the DNA/RNA binding shown by our study.

## 4. Materials and Methods

### 4.1. Reagents

ctDNA and poly A-poly U were purchased from Sigma-Aldrich (Merck, Europe) and used without further purification. The stock solution was prepared by dissolved a small portion of the lyophilized polynucleotide in 200 μL of NaCac 50 mM buffer and stored at 5 °C overnight. The concentration of the stock solution was checked by measuring the absorption of four accumulative additions of the stock solution to a 1 mL of NaCac 50 mM. Concentration was then calculated by using the molar extinction coefficient given by the supplier (6600 M^−1^ cm^−1^ for ctDNA and 6000 M^−1^ cm^−1^ for poly A-poly U). Stock solutions were maintained at −10 °C and defrost before use. Ethidium bromide (EB) (3,8-diamino-5-ethyl-6-phenylphenanthridinium bromide) and DAPI (2-(4-amidinophenyl)-6-indolecarbamidine dihydrochloride, 4′,6-diamidino-2-phenylindole dihydrochloride) were purchased from commercial sources and used without further purification.

### 4.2. Synthesis of Ligands

Synthesis of the ligands 6-[7-(diaminoethyl)-3,7-diazaheptyl]-3,6,9-triaza-1-(2,6-pyridina)cyclodecaphane (**L1**) and 6-[6′-[3,6,9-triaza-1-(2,6-pyridina)cyclodecaphan-6-yl]-3-azahexyl]-3,6,9-triaza-1-(2,6-pyridina)cyclodecaphane (**L2**) was carried out following the general procedures described in the literature [20].

### 4.3. EMF Measurements

The potentiometric titrations were carried out at 298.1 ± 0.1 K using NaCl 0.15 M as supporting electrolyte (Merck, Europe). The acquisition of the emf data was performed with the computer program PASAT. The reference electrode was an Ag/AgCl electrode in saturated KCl solution. The glass electrode was calibrated as a hydrogen-ion concentration probe by titration of previously standardized amounts of HCl with CO_2_-free NaOH solutions and the equivalent point determined by the Gran’s method, which gives the standard potential, E°’, and the ionic product of water (pK_w_ = 13.73(1)) [23,24].

The computer program HYPERQUAD was used to calculate the protonation and stability constants [25]. The pH range investigated was 2.0–11.0, and the concentration of the metal ions and the ligands ranged from 1 × 10^−3^ to 5 × 10^−3^ M with M:L molar ratios varying from 2:1 to 1:2. The different titration curves for each system (at least two) were treated either as a single set or as separated curves without significant variations in the values of the stability constants. Finally, the sets of data were merged together and treated simultaneously to provide the final stability constants.

### 4.4. Thermal Melting Experiments

Thermal melting curves were measured on an Agilent 8453 spectrometer equipped with a Peltier temperature controller system (±0.1 °C). The ratio of nucleic acid to compound used ranges from 20:1 to 2:1. Thermal melting curves were determined by following the absorption change at 260 nm in 50 mM cacodylate buffer (pH 7.4) as a function of temperature in the absence or presence of polyamine derivatives. The absorbance of the ligand was subtracted from every curve, and the absorbance scale was normalized. The *T*_m_ values were taken as the midpoints of the transition curves as determined from the maximum of the first derivative. Δ*T*_m_ values were calculated by subtracting *T*_m_ for the free nucleic acid from *T*_m_ for the complex [22]. Every Δ*T*_m_ value reported here was the average of at least two measurements; the error in Δ*T*_m_ was ±0.5 °C.

### 4.5. Fluorescent Indicator Displacement Assays

Displacement experiments were recorded on a PTI spectrofluorimeter in the 540−680 nm range for EB and 430–700 nm range for DAPI with an excitation wavelength of 520 nm and 400 nm, respectively. The concentration of polynucleotide ranges from 3 to 4 × 10^−5^ M for EB displacement assays and 1 to 2 × 10^−5^ M for DAPI displacement assays. The fluorescence was normalized by the maximum fluorescence signal when the marker was bound to the nucleic acid in the absence of competition for binding and was corrected for background fluorescence of the free marker in solution.

### 4.6. Theoretical Studies

The generation of the computational models for the proposed complexes was performed using the density functional theory computational method as well as the Becke three-parameter Lee-Yang-Parr hybrid functional (B3LYP) [26,27,28]. All the gas-phase optimizations were carried out using the Ahlrichs’ basis set def2-SV(P) [29] for all atoms except for Zn^2+^ for which it was employed the MDF10 Stuttgart-Dresden effective core potential [30]. The influence of the dispersion was also taken into account by means of the Grimme’s dispersion (*IOp(3/124 = 30)*) correction [31]. Computations were carried out using the program Gaussian09 C.01. [32], gMolden [33], and PyMOL [34] were used for visual inspection and to create the molecular graphics. Finally, in order to use the obtained complexes for the MD and QM/MM-MD modeling, additional parameters were calculated using the MCPB parameter builder [35]. Briefly, the force field parameters (bond, angle, dihedral, electrostatic, van der Waals terms) were obtained at the B3LYP/def2-SV(P) level of theory, and the ESP punctual charges were derived by fitting the electrostatic potential according to the MDF10 Stuttgart-Dresden scheme.

The interaction studies of the Zn^2+^ complexes with the nucleic acids were performed through a QM/MM-MD approach. For the building of the double helix of calf thymus DNA systems, a representative duplex B-type DNA was taken from the Protein Data Bank (PDB, ID 1BNA) as a starting structure [36,37]; while the RNA one was build using the 3D-NuS program due to the absence of crystalline structures [38]. Once the duplex DNA/RNA systems were built, the ligand/complex systems were approximated, and, finally, the resulting system was solvated with the addition of 4258 water residues using TIP3PBOX and neutralized. Then, each system was energetically minimized and, after an equilibration stage at 300 K, a total of 20 ps molecular dynamics (MD) was performed. All the studies have been performed using AMBER16 [39] (Assisted Model Building with Energy Refinement) software. The organic compounds have been modeled using the *gaff* [40] force field, while the *ff12SB* force field was used for the nuclear bases. After the MD modeling, a final QM/MM-MD study was carried out at 300 K for 3 ns. The external Gaussian package was used for the quantum mechanical calculations. The QM region encompassed the complex, whereas the MM region included all the remaining solvent, counterions, and poly-nucleic strands. Therefore, no covalent bond had been cut when defining the QM/MM-MD boundary. The inner QM region was treated at the B3LYP-D3/def2-SV(P) level of theory. A cutoff of 10 Å was employed for the evaluation of the electrostatic interactions within the MM-MD region and of 10 Å for the QM region. The MD and QM/MM-MD simulation trajectories were analyzed using the cpptraj [41] module within AmberTools17 and PyMOL was used for visual inspection and to create the molecular graphics.

### 4.7. MTT Assay

T24 and 253J transitional-cell human bladder carcinoma cells and VERO green monkey kidney cells were cultured at 37 °C in a 5% CO_2_ in air atmosphere. VERO cells were grown in Eagle’s minimum essential medium and T24 and 253J in McCoy’s 5A media supplemented with 10% FBS, 2 mM *L*-glutamine, 100 U/mL penicillin, and 100 μg/mL streptomycin. Cells were seeded at 2.5 × 10^4^ cells/mL in 96-well plates and treated with **L1**, **L2**, and their metal complexes at different concentrations (10, 50, and 100 μM) for 48 h.

The MTT assay is based on the ability of mitochondrial dehydrogenases of viable cells to reduce MTT to a purple formazan product (insoluble in water), which can be quantified spectrophotometrically (after solubilization in DMSO). For each well, the culture medium was aspirated, and 100 µL of MTT solution (5 mg/mL in PBS) was added to the cells, which were incubated for 4 h at 37 °C. After this period, the formazan crystals formed were dissolved upon the addition of 100 µL of DMSO, and the absorbance of these solutions was measured at 570 nm (against a blank containing MTT and DMSO in a 1:1 ratio). The results were expressed as the percentage of MTT reduction, assuming the absorbance of control cells as 100%.

## 5. Conclusions

In summary, the interaction of two polyamine-based ligands and their Zn^2+^ metal complexes with duplex DNA and RNA was evaluated using UV thermal and fluorescence indicator displacement assays in combination with theoretical studies. Both ligands show a high affinity for ds-DNA/RNA and selectivity for ds-RNA. The ability to interact with these duplexes was blocked upon Zn^2+^ coordination, which was confirmed by the low variation in the melting temperature and poor displacement of the fluorescent dye from the ds-DNA/RNA. All the experimental findings were supported using molecular modeling, which indicates that a large number of electrostatic contacts for the free ligands are hampered upon metal ion coordination to the nitrogen atoms of the ligands. Finally, cell viability assays show a decrease in the cytotoxicity of the metal complexes in comparison with free ligand, which can be associated with the observed binding to ds-DNA/RNA.

## Figures and Tables

**Table 1:** IC_50_ values determined for the displacement of EB and DAPI from ctDNA and poly A-poly U by addition of **L1** and **L2** (NaCac 50 mM, pH 7.4).

		EB	DAPI
**L1**	ctDNA	1.37	
	poly A-poly U	0.63	0.85
**L2**	ctDNA	2.03	
	poly A-poly U	0.86	0.45

**Table 2:** Δ*T*_m_ values determined for the metal complexes of **L1**–**L2** with ctDNA (r = 0.05, NaCac 50 mM, pH 7.4).

	Free Ligand	1:1 (Zn^2+^–L)	2:1 (Zn^2+^–L)
**L1**	21.3	1.9	1.1
**L2**	17.1	4.1	−1.1

## Data Availability

The datasets generated during the current study are available from the corresponding author on reasonable request.

## Supplementary Materials

The following are available online. Figures S1–S8, Tables S1–S5. Tables S1 and S2 gather the logarithms of the stepwise protonation constants of **L1** and **L2** determined and equilibrium constants for the interaction of Zn^2+^ with **L1** and **L2**. Tables S3 and S4 collect the structural data for complex Zn2**L1** and Zn2**L2** and Table S5 the Δ*Tm* values determined for the interaction of **L1** and **L2** toward ds-DNA and ds-RNA. Figures S1–S3 collect the distribution diagrams for **L1** and **L2** and the metal complex systems Figures S4–S6 gather the representation of the interaction of ligands and metal complexes with ds-DNA and ds-RNA.

## Sample Availability

Samples of the compounds are available from the authors on reasonable request and depending on the stock.
