# Supplementary material for: Ditopic Aza-Scorpiand Ligands Interact Selectively with ds-RNA and Modulate the Interaction upon Formation of Zn2+ Complexes
Source: Molecules. 2021 Jun 28;26(13):3957. doi: 10.3390/molecules26133957 (PMC8272215; doi:10.3390/molecules26133957)
Supplement: Supplementary file 1 [file molecules-26-03957-s001.zip › molecules-1246874-supplementary.pdf]

Supporting Information

Ditopic Aza-Scorpiand Ligands Interact Selectively with ds-RNA and Modulate the Interaction Upon Formation of Zn^{2+} Complexes

Lluís Guijarro [1], Álvaro Martínez-Camarena [1], Javier U. Chicote [2,3], Antonio García-España [2], Enrique García-España [1,*], Mario Inclán [1], Begoña Verdejo,[1,*] and Jorge González-García [1,*]

[1] Instituto de Ciencia Molecular (ICMol), Departamento de Química Inorgánica, Universidad de Valencia. C/ Catedrático José Beltrán 2, 46980 Paterna, Valencia, Spain; lluis.guijarro@uv.es (L.G.); alvaro.martinez@uv.es (Á.M.-C.); mario.inclan@uv.es (M.I.)

[2] Institut d'Investigació Sanitària Pere Virgili (IISPV), Centro de I+D+I en Nutrición y Salud Avda. de la Universitat 1, E-43204 Reus, Tarragona, Spain; jugarte@piushospital.cat (J.U.C.); antoniogem85@gmail.com (A.G.-E.)

[3] Pathology Unit, Joan XXIII University Hospìtal, C/ Dr. Mallafrè Guasch 4, 43005 Tarragona, Spain

* Correspondence: jorge.gonzalez@uv.es (J.G.-G.); begona.verdejo@uv.es (B.V.); enrique.garcia-es@uv.es (E.G.-E.)

Table of contents

Table S1. Logarithms of the stepwise protonation constants for the protonation of **L1** and **L2** determined at 298.1 K in 0.15 M NaCl.

Table S2. Logarithms of the equilibrium constants for the interaction of Zn^{2+} with **L1** and **L2** determined at 298.1 K in NaCl 0.15 M.

Table S3. Structural data for complex Zn_2**L1**

Table S4. Structural data for complex Zn_2**L2**

Table S5. ΔT_m values determined for the interaction of **L1** and **L2** towards ctDNA and polyA – polyU at different *r*. (p): precipitated.

Figure S1. Distribution diagrams for **L1** (top panel) – **L2** (bottom panel). [L] = 10^{-3} M.

Figure S2. Distribution diagram for the system Zn^{2+}-**L1** (top panel), Zn^{2+}-**L2** (bottom panel), [L]=10^{-3} M, [Zn^{2+}] = 10^{-3} M.

Figure S3. Distribution diagram for the system Zn^{2+}-**L1** (top), Zn^{2+}-**L2** (bottom), [L]=10^{-3} M, [Zn^{2+}] = 2 x 10^{-3} M.

Figure S4. Representation of the interaction of **L2** with the ds-DNA, poly-nucleotide surface of **L2** with the ds-DNA and deviation of the ds-DNA structure after interaction regarding its original conformation.

Figure S5. Representation of the interaction of **L2** with the ds-RNA, poly-nucleotide surface of **L2** with the ds-RNA and deviation of the ds-RNA structure after interaction regarding its original conformation.

Figure S6. Representation of the interaction of Zn_2**L1**$^{4+}$ with the ds-RNA, poly-nucleotide surface of Zn_2**L1**$^{4+}$ with the ds-RNA and deviation of the ds-RNA structure after interaction regarding its original conformation.

Figure S7. Representation of the interaction of Zn_2**L2**$^{4+}$ with the ds-DNA, poly-nucleotide surface of Zn_2**L2**$^{4+}$ with the ds-DNA and deviation of the ds-DNA structure after interaction regarding its original conformation.

Figure S8. Representation of the interaction of Zn_2**L2**$^{4+}$ with the ds-RNA, poly-nucleotide surface of Zn_2**L2**$^{4+}$ with the ds-RNA and deviation of the ds-DNA structure after interaction regarding its original conformation.

Table S1. Logarithms of the stepwise protonation constants for the protonation of **L1** and **L2** determined at 298.1 K in 0.15 M NaCl.

**Reaction**[a]	**L1**	**L2**
$H + L \rightleftarrows HL$	9.84(1)[b]	10.19(3)
$H + HL \rightleftarrows H_2L$	9.72(3)	9.58(2)
$H + H_2L \rightleftarrows H_3L$	8.99(2)	8.74(3)
$H + H_3L \rightleftarrows H_4L$	8.54(1)	8.26(2)
$H + H_4L \rightleftarrows H_5L$	7.36(2)	7.28(3)
Log ß[c]	44.46(2)	44.03(3)

[a] Charges omitted for clarity. [b] Values in parentheses are standard deviation in the last significant figure. [c] $\log\beta^{c} = \sum \log K$. [d] Reference 1

Table S2. Logarithms of the equilibrium constants for the interaction of Zn^{2+} with **L1** and **L2** determined at 298.1 K in NaCl 0.15 M.

**Reaction**[a]	**L1**	**L2**
$ZnH_2L + H \rightleftarrows ZnH_3L$	5.36(1)[b,c]	-
$ZnHL + H \rightleftarrows ZnH_2L$	8.44(3)	8.35(5)
$ZnL + H \rightleftarrows ZnHL$	9.80(6)	9.68(5)
$Zn + L \rightleftarrows ZnL$	15.99(6)	17.23(7)
$ZnL + H_2O \rightleftarrows ZnL(OH) + H$	-10.72(7)	-10.29(9)
$2Zn + L \rightleftarrows Zn_2L$	23.88(4)	28.93(3)
$ZnL + Zn \rightleftarrows Zn$	7.89(5)	11.70(8)
$Zn_2L + H_2O \rightleftarrows Zn_2L(OH) + H$	-8.77(5)	-8.76(7)
$Zn_2L(OH) + H_2O \rightleftarrows Zn_2L(OH)_2 + H$	-11.11(8)	-10.0(1)
$2Zn + L + H_2O \rightleftarrows Zn_2L(OH) + H$	15.10(5)	20.17(5)
$2Zn + L + 2H_2O \rightleftarrows Zn_2L(OH)_2 + 2H$	3.99(8)	10.19(6)

[a] Charges omitted for clarity. [b] Values in parentheses are standard deviation in the last significant figure. [c]Reference 1

Table S3. Structural data for complex Zn_2**L1**

Bond distances (Å)			**Bond angles (deg.) 1st complex**				**Bond angles (deg.) 2nd complex**			
N1	Zn1	2.057	N1	Zn1	N2	79.3	N5	Zn2	N6	109.3
N2	Zn1	2.207	N1	Zn1	N3	93.3	N5	Zn2	N7	101.8
N3	Zn1	2.422	N1	Zn1	N4	79.2	N5	Zn2	N8	121.8
N4	Zn1	2.212	N1	Zn1	O1	88.5	N5	Zn2	O3	94.9
O1	Zn1	2.174	N1	Zn1	O2	174.5	N6	Zn2	N7	82.6
O2	Zn1	2.146	N2	Zn1	N3	80.9	N6	Zn2	N8	128.7
N5	Zn2	2.140	N2	Zn1	N4	151.0	N6	Zn2	O3	89.0
N6	Zn2	2.115	N2	Zn1	O1	99.3	N7	Zn2	N8	83.5
N7	Zn2	2.215	N2	Zn1	O2	102.6	N7	Zn2	O3	163.0
N8	Zn2	2.104	N3	Zn1	N4	81.0	N8	Zn2	O3	90.4
O3	Zn2	2.249	N3	Zn1	O1	178.1				
			N3	Zn1	O2	92.0				
			N4	Zn1	O1	9.5				
			N4	Zn1	O2	100.5				
			O1	Zn1	O2	86.1				

Table S4. Structural data for complex Zn_2**L2**

Bond distances (Å)			**Bond angles (deg.) 1st complex**				**Bond angles (deg.) 2nd complex**			
N1	Zn1	2.082	N1	Zn1	N2	78.0	N6	Zn2	N7	79.2
N2	Zn1	2.269	N1	Zn1	N3	94.3	N6	Zn2	N8	97.5
N3	Zn1	2.190	N1	Zn1	N4	78.2	N6	Zn2	N9	78.8
N4	Zn1	2.274	N1	Zn1	N5	175.8	N6	Zn2	O2	89.4
N5	Zn1	2.241	N1	Zn1	O1	93.4	N6	Zn2	O3	166.6
O1	Zn1	2.245	N2	Zn1	N3	82.5	N7	Zn2	N8	84.0
N6	Zn2	2.060	N2	Zn1	N4	150.4	N7	Zn2	N9	152.8
N7	Zn2	2.207	N2	Zn1	N5	99.3	N7	Zn2	O2	100.2
N8	Zn2	2.274	N2	Zn1	O1	98.1	N7	Zn2	O3	106.3
N9	Zn2	2.220	N3	Zn1	N4	82.1	N8	Zn2	N9	83.2
O2	Zn2	2.307	N3	Zn1	N5	81.9	N8	Zn2	O2	172.5
O3	Zn2	2.172	N3	Zn1	O1	172.2	N8	Zn2	O3	95.2
			N4	Zn1	N5	103.3	N9	Zn2	O2	95.3
			N4	Zn1	O1	100.6	N9	Zn2	O3	98.6
			N5	Zn1	O1	90.1	O2	Zn2	O3	77.6

Table S5. ΔT_m values determined for the interaction of **L1** and **L2** towards ctDNA and polyA – polyU at different *r*. (p): precipitated.

				r			
		**free**	**0.5**	**0.2**	**0.1**	**0.05**	**0.01**
**L1**	ct-DNA	80.3	(p)	3.2	1.9	0.7	0.1
	polyA - polyU	52.0	(p)	26.5	22.0	21.3	0.5
**L2**	ct-DNA	-	1.6	1.2	1.0	1.3	1.4
	polyA - polyU	-	24.7	21.4	16.9	17.1	0.2

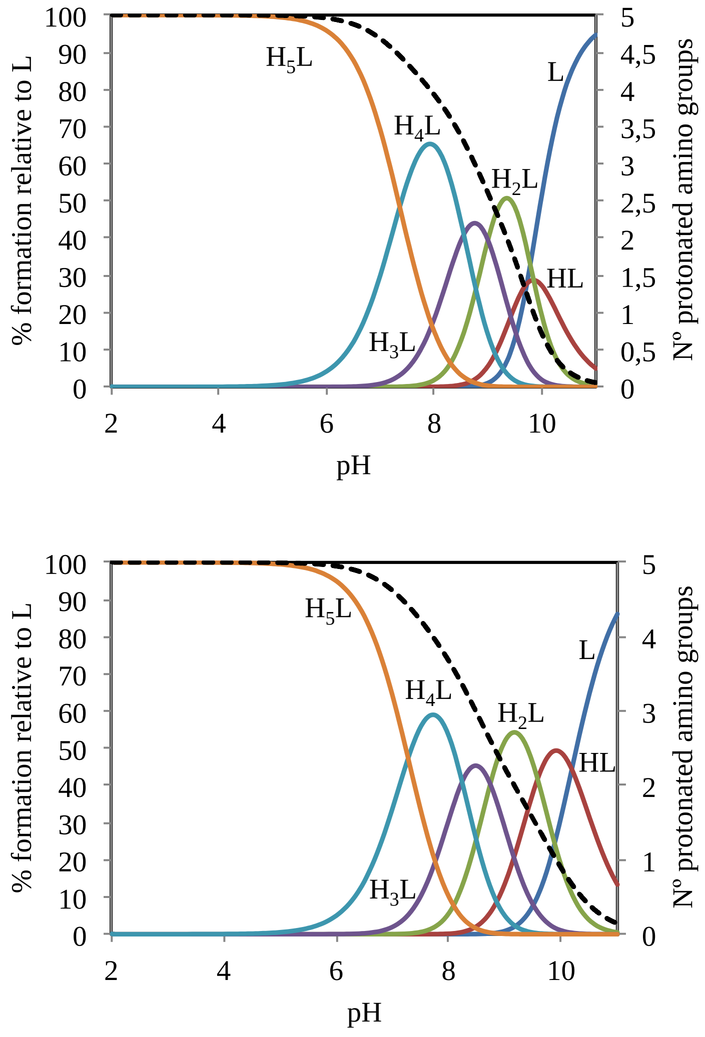

Figure S1. Distribution diagrams for **L1** (top panel) – **L2** (bottom panel). [L] = 10^{-3} M.

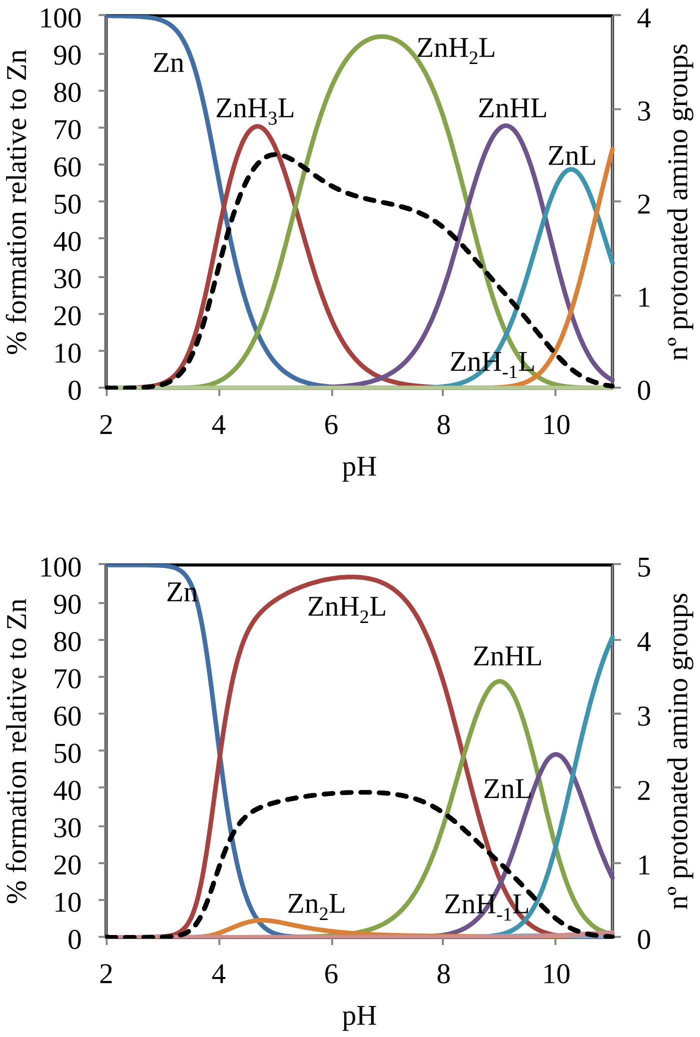

Figure S2. Distribution diagram for the system Zn^{2+}-**L1** (top panel), Zn^{2+}-**L2** (bottom panel), $[L]=10^{-3}$ M, $[Zn^{2+}] = 10^{-3}$ M.

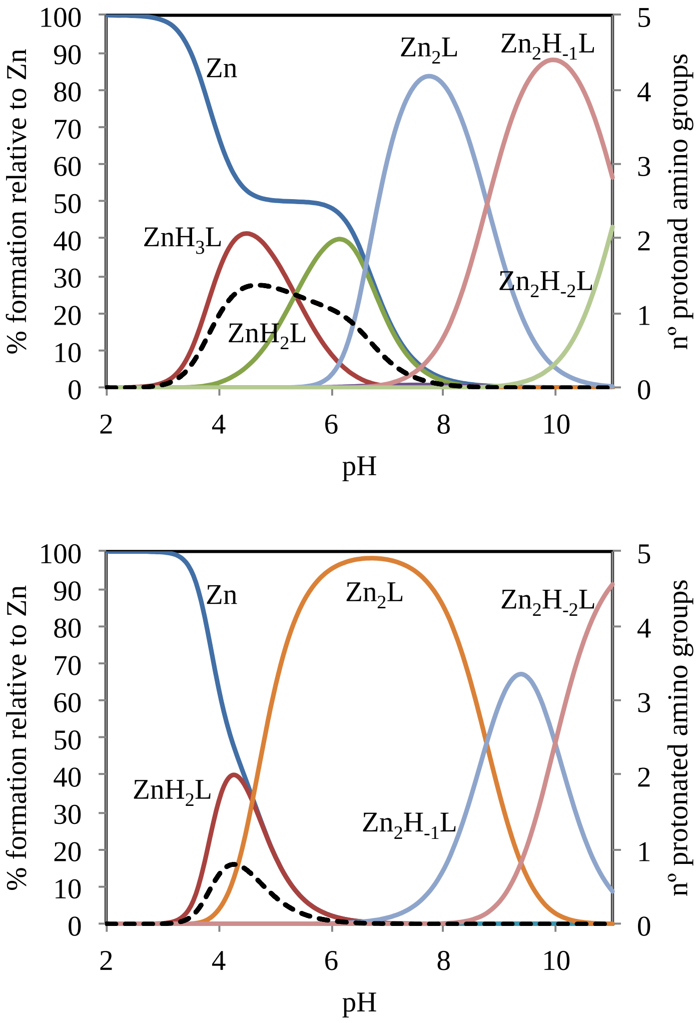

Figure S3. Distribution diagram for the system Zn^{2+}-**L1** (top), Zn^{2+}-**L2** (bottom), [L]=10^{-3} M, $[Zn^{2+}]$ = 2 x 10^{-3} M.

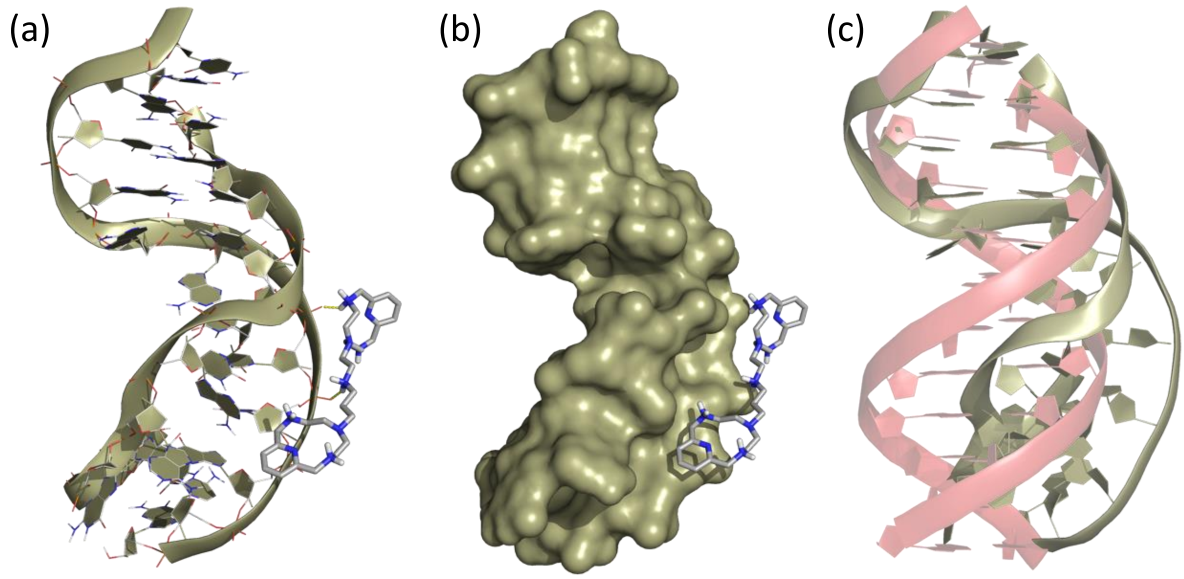

Figure S4. Representation of the interaction of **L2** with the ds-DNA (a), poly-nucleotide surface of **L2** with the ds-DNA (b) and deviation of the ds-DNA (c) structure after interaction regarding its original conformation (represented in red).

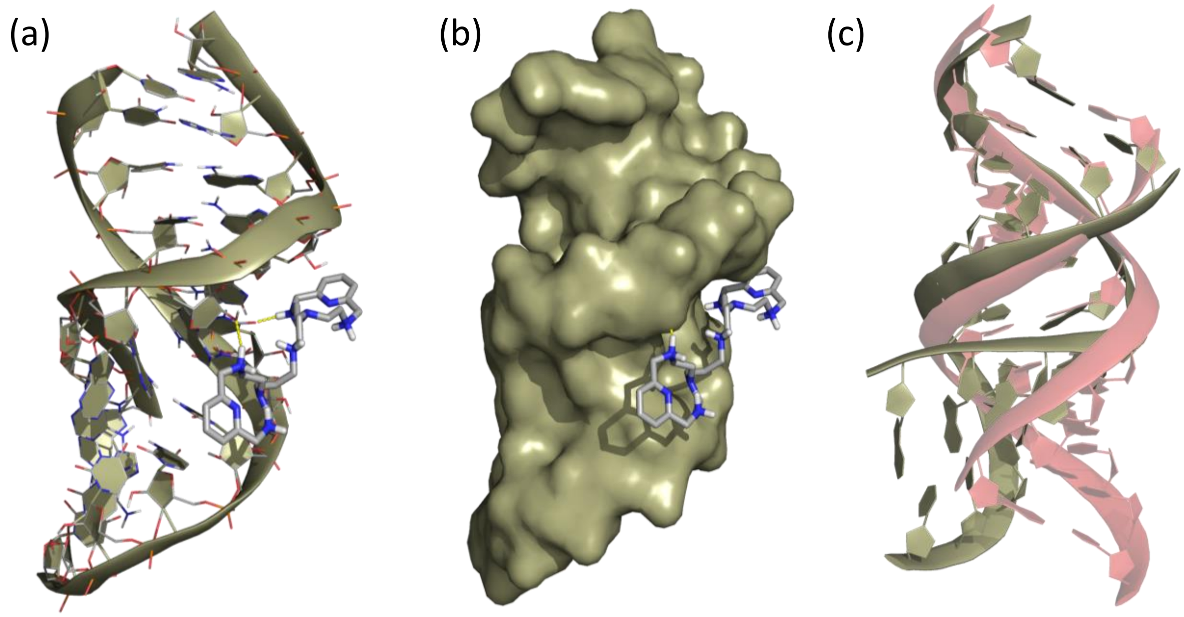

Figure S5. Representation of the interaction of **L2** with the ds-RNA (a), poly-nucleotide surface of **L2** with the ds-RNA (b) and deviation of the ds-RNA (c) structure after interaction regarding its original conformation (represented in red).

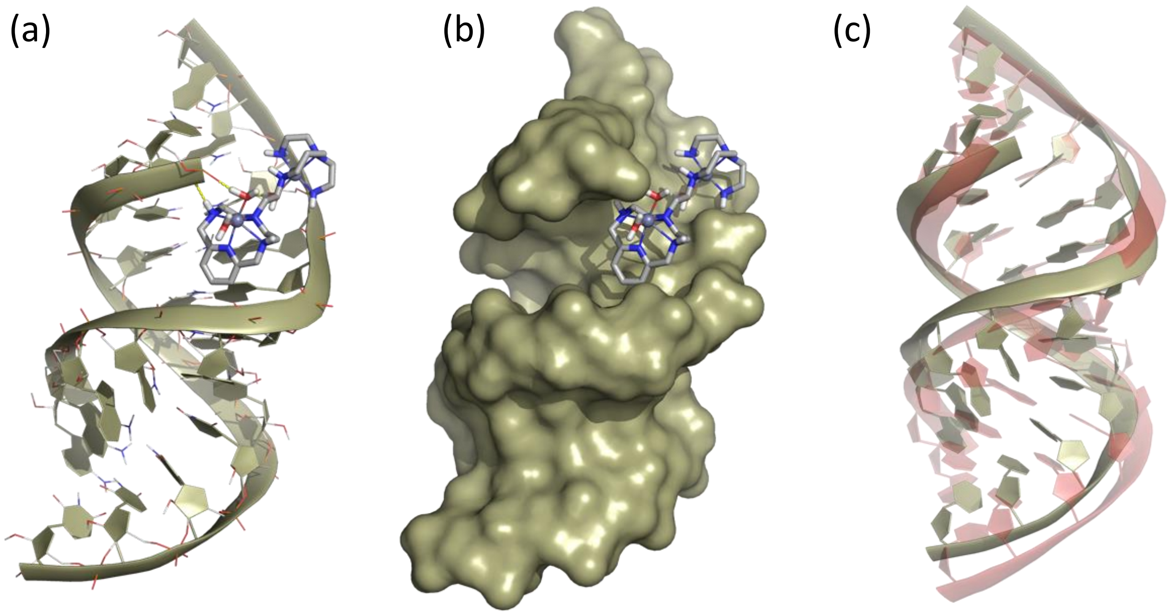

Figure S6. Representation of the interaction of $Zn_2\mathbf{L1}^{4+}$ with the ds-RNA (a), poly-nucleotide surface of $Zn_2\mathbf{L1}^{4+}$ with the ds-RNA (b) and deviation of the ds-RNA (c) structure after interaction regarding its original conformation (represented in red).

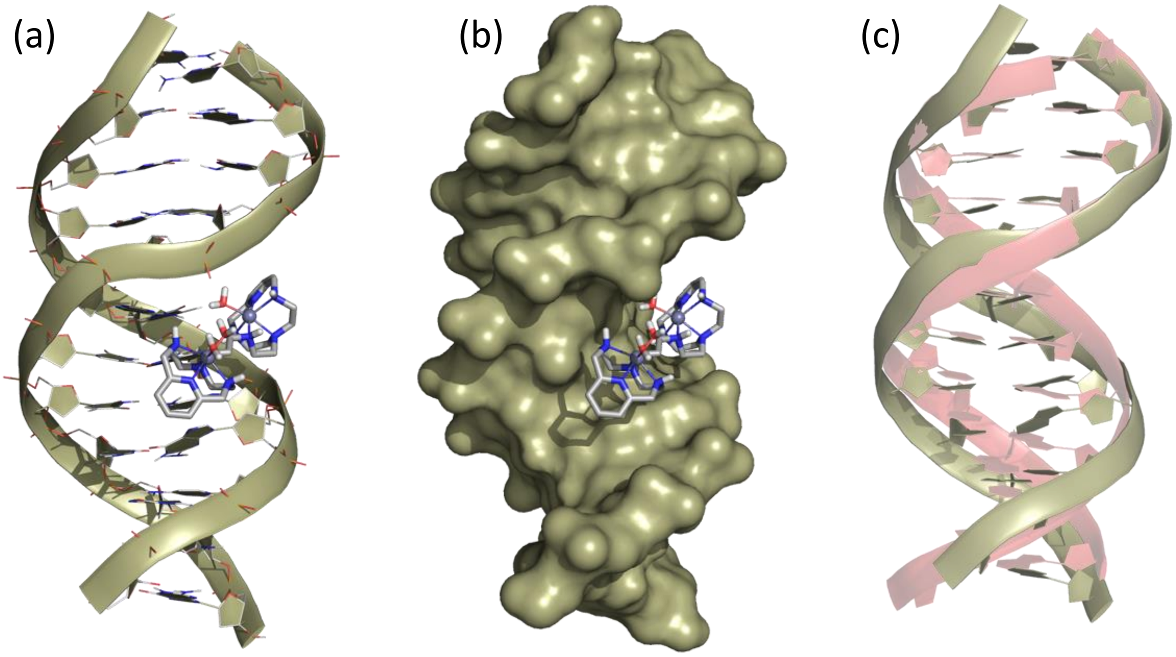

Figure S7. Representation of the interaction of $Zn_2\mathbf{L2}^{4+}$ with the ds-DNA (a), poly-nucleotide surface of $Zn_2\mathbf{L2}^{4+}$ with the ds-DNA (b) and deviation of the ds-DNA (c) structure after interaction regarding its original conformation (represented in red).

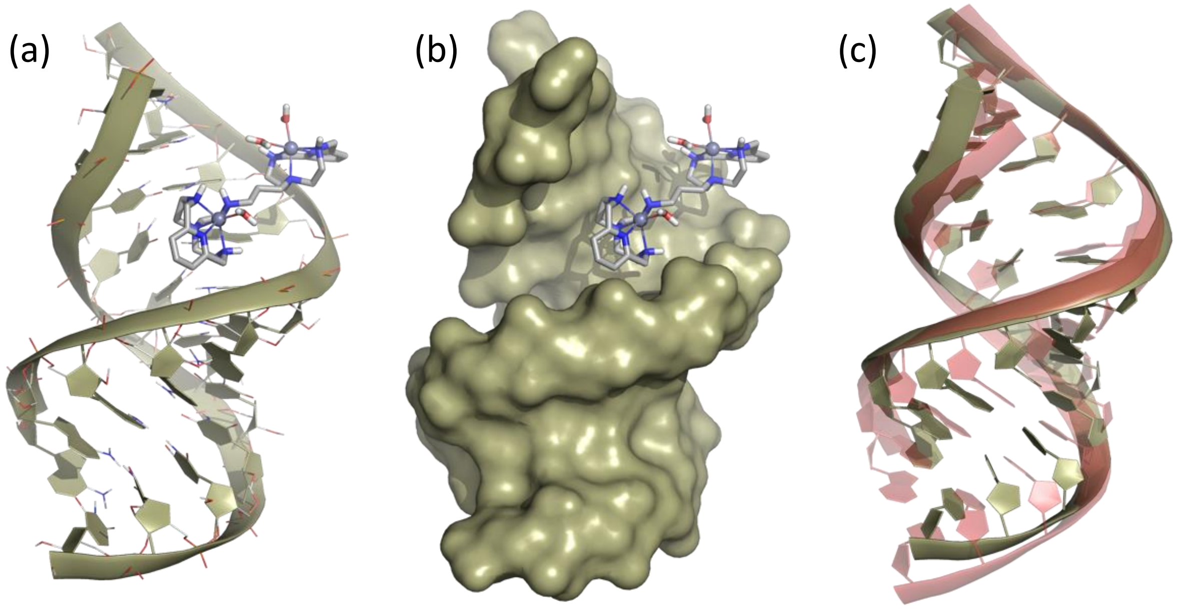

Figure S8. Representation of the interaction of Zn_2**L2**$^{4+}$ with the ds-RNA (a), poly-nucleotide surface of Zn_2**L2**$^{4+}$ with the ds-RNA (b) and deviation of the ds-DNA (c) structure after interaction regarding its original conformation (represented in red).

[1] Guijarro, L.; Inclan, M.; Pitarch-Jarque, J.; Domenech-Carbo, A.; Chicote, J.U.; Trefler, S.; García-España, E., García-España, A.; Verdejo, B. Homo- and Heterobinuclear Cu2+ and Zn2+ Complexes of Ditopic Aza Scorpiand Ligands as Superoxide Dismutase Mimics. *Inorg. Chem.*, **2017**, *56*, 13748–13758.
